# Cost-effective smartphone-based method for low range chemical oxygen demand analysis

**DOI:** 10.1016/j.mex.2023.102300

**Published:** 2023-07-26

**Authors:** Inalmar D. Barbosa Segundo, Jussara C. Cardozo, Pollyana Souza Castro, Amanda D. Gondim, Elisama V. dos Santos, Carlos A. Martínez-Huitle

**Affiliations:** a Renewable Energies and Environmental Sustainability Research Group, Institute of Chemistry, Federal University of Rio Grande do Norte, Campus Universitário, Av. Salgado Filho 3000, Lagoa Nova, Natal, RN CEP 59078-970, Brazil; b Institute of Chemistry, Federal University of Rio Grande do Norte, Av. Salgado Filho 3000, Lagoa Nova, Natal, RN CEP 59078-970, Brazil; c National Institute for Alternative Technologies of Detection, Toxicological Evaluation and Removal of Mi-cropollutants and Radioactives (INCT–DATREM), Institute of Chemistry, UNESP, P.O. Box 355, Araraquara, SP 14800-900, Brazil

**Keywords:** COD, Smartphone-based technology, HSV image processing, Digital image colorimetry, *Smartphone-based method for low range chemical oxygen demand analysis*

## Abstract

Aiming the decentralization of monitoring policies and to facilitate the work of researchers, mainly in developing countries, the present method deals with the explanation of a simple and rapid protocol for chemical oxygen demand (COD) analysis through the use of digital smartphone devices coupled with a camera and a free app available for Android operating system that recognizes HSV (hue, saturation, value). The calibration of the method is done based on the theoretical values of potassium hydrogen phthalate for a proper and reliable build of the calibration curve by using the smartphone-based technique and the digested samples of COD. The coefficient of determination (R^2^) attained a value upper than 0.99, providing a high confidence levels, and the method achieved 97% of average accuracy in samples with COD values ranging from 0 to 150 mg L^−1^. Finally, the procedure here presented can be a great support for scientific laboratories and monitoring policies, once it can efficiently substitute expensive spectrophotometers and can improve and ensure the sustainable management of water sanitation, which is one of the sustainable goals proposed by the United Nations.

• COD measurements, based on the use of a simple smartphone with a camera, can be a promising way for environmental analysis when spectrophotometers are not available, such as decentralized approaches.
• The use of smartphone protocol is a novel initiative to fulfill sustainable development goal 6 on clean water and sanitation.
• The smartphone is capable to read the difference of HSV values efficiently and can substitute the use of expensive spectrophotometers.

## Graphical abstract

**Figure:**
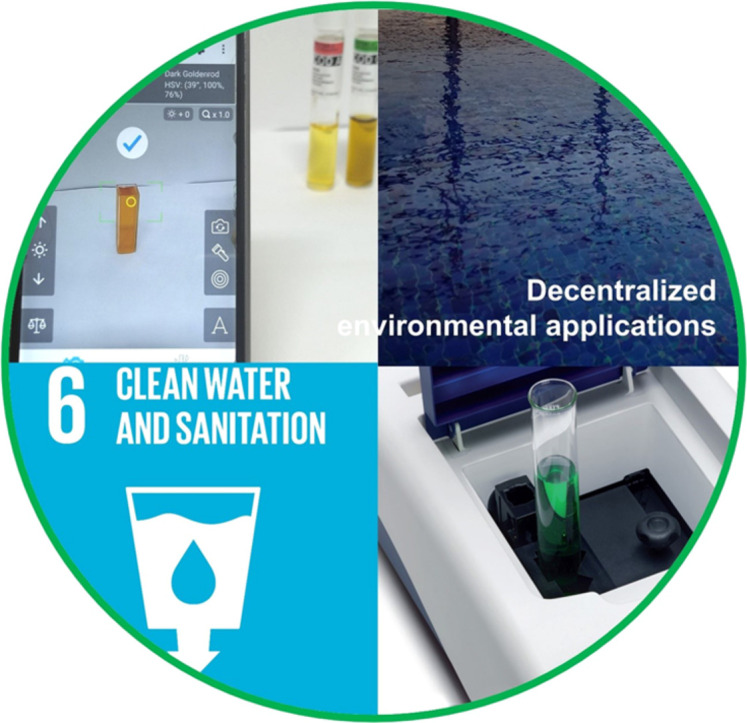

Specifications table

**Table:** 

Subject area:	*Environmental Science*
More specific subject area:	*Water and wastewater quality*
Name of your method:	*Smartphone-based method for low range chemical oxygen demand analysis*
Name and reference of the original method:	*C. M. de Castro, P. Olivi, K. C. de F. Araújo, I. D. Barbosa Segundo, E. V. dos Santos, C. A. Martínez-Huitle. Environmental application of a cost-effective smartphone-based method for COD analysis: Applicability in the electrochemical treatment of real wastewater, Sci. Tot. Env. 855. (2023).*[1] https://doi.org/10.1016/j.scitotenv.2022.158816.
Resource availability:	*Color Grab (color detection) app*

## Introduction

Chemical oxygen demand (COD) is a measure that indicates the amount of available oxygen that can be consumed by reactions in a determined solution, and it is commonly expressed as the mass of oxygen consumed over the volume of solution (mg L^−1^). The COD analysis is widely used to quantify the number of organics in water since it can be indirectly responsible to show the amount of oxidizable pollutants found in the most diverse waterbodies and wastewaters [2]. It is essential in terms of water policy by providing a metric that can predict the effect an effluent will have on the receiving body, much like biochemical oxygen demand (BOD), but faster and developed to be also a means of measuring industrial or trade wastewaters, once it can oxidize matter that is generally inert to biological action, with the result that its value for wastewaters is greater than that for BOD [3].

Basically, the methods available for COD analysis induce the sample to be oxidized in contact with a highly oxidizing mix of potassium dichromate and sulphuric acid, and the reaction extention can be colorimetrically assessed. This colorimetric difference is commonly analyzed by using spectrophotometers in specific wavelengths; however, the high availability and decentralizing access of smartphone devices coupled with the advances in the technology employed in their cameras increased the interest in applying it to analytical purposes. The smartphone cameras can make the process faster, simple, and lower cost, despite generating immediate results [4], and have gained visibility through their use in several areas of science [5], [6], [7], [8].

The facility of the method gives a rapid and simple procedure to substitute expensive, big, and heavy spectrophotometers [9], only for a while when the equipment is broken or unusable, or even definitely, once the method proves to be reliable since it shows a linear behavior with the yellowish color of the digested samples of low range COD, uncommitted to the matrix of the waters/wastewaters.

It is also good to mention that most of the spectrophotometers are not compatible with the commercial kits developed by analytical companies, and specific spectrophotometers are necessary to analyze the samples. In this frame, with the proposed procedure detailed here, these equipments are not more necessary, which contributes to the universalization of science and governmental policy as well as the digitalization.

## Method details

This method is based on developing a calibration curve of potassium hydrogen phthalate standard solutions through using a smartphone camera with a specific app that transform the images of the samples in HSV (hue, saturation, value) values. In fact, the saturation (S) of the captured image shows a linear behavior that can be plotted and used for COD quantification because it represents the color intensity and its purity degree through the radial coordinate. This procedure permits to determine the COD results without the necessity of spectrophotometers, which have high associated costs for their adquisition and maintenance.

### Apparatus

Translucent glass vials for sample storage and analysis (COD tubes)

Digestor for the thermal treatment of the COD tube vials

Smartphone with Android as the operating system, for the Color Grab app installation, with a camera in good functioning conditions

Analytical balance to weigh potassium hydrogen phthalate

Beakers and volumetric flasks to solubilize and prepare the potassium hydrogen phthalate standard solutions

Ruler

White background

Artificial light

### Reagents

Kit of COD tube tests by HANNA (low range, 0 – 150 mg L^−1^, Reference n. HI93754F-25)

Potassium hydrogen phthalate of analytical grade

### Procedure

To determine the COD of the samples, it is necessary to use a calibration curve with a reference standard. Thus, before starting the analysis, the external calibration curve must be constructed according to the following protocol. Known concentration solutions of potassium hydrogen phthalate (KHP) are used for this purpose. The theoretical relationship between KHP and COD is 1 mg of KHP = 1.171 mg COD.

Firstly, a stock solution of 500 mg KHP L^−1^ is prepared (250 mg of KHP are solubilized in 500 mL of distilled water), from which all the other standard solutions are prepared (Table 1). The correct weighing of the KHP for the stock solution and the volume transferred from the stock solution to the volumetric flasks are essential to guarantee the accuracy and efficacy of the method.

**Table 1:** Procedure and values of theoretical COD for KHP standard solutions.

Pipetted volume from the stock solution (mL)	Volumetric flask (mL)	KHP Standards (mg C_8_H_5_KO_4_ L^−1^)	Theoretical COD (mg O_2_ L^−1^)
12	50	120	140.5
10.5	50	105	123.0
9	50	90	105.4
7.5	50	75	87.8
6	50	60	70.3
4.5	50	45	52.7
3.0	50	30	35.1
1.5	50	15	17.6
Distilled water		0	0.0

50 mL is much more than necessary for the filling of the glass vials where the standard solutions are analyzed but this procedure is strongly recommended because facilitates the volume to be transferred from the stock solution. Besides, it is appropriate to carried out as least a duplicate of each sample, for statistical purposes and a good validation of the method [10,11].

The preparation of the COD standard solutions can vary with the utilization of different methods, but the yellowish character of the samples in low-range COD concentration is always the same and can be analyzed by the color saturation. 2 mL standard solutions were transferred to the HANNA COD tube tests (from 0 to 150 mg L^−1^), digested at 150 °C for 2 h, and cooled at room temperature. This volume is necessary to perform the COD analysis following the HANNA tests; however, the procedure can be different once there are other methods, as the widely known 5220 from the Standards Methods for the Examination of Water and Wastewater [12]. Thus, the present method is valid for the analysis of all the low-range COD procedures that evolve the changing in color during the digestion of K_2_Cr_2_O_7_, due to the reduction of chromium (VI) to chromium (III). It can efficiently quantify the linearity of different standards by the saturation of the color samples, making it unnecessary using UV light absorption at specific wavelengths. It is important to point out that the concentration investigated by the method ranges from 0 to 150 mg L^−1^, once higher than this range concentrations makes it impossible to verify the difference in the color saturation of the samples, since it achieves 100% with all the standards, hindering the possibility to use the method. The samples that are expected to surpass the superior range concentration (150 mg O_2_ L^−1^ of COD values), should be diluted.

Before analyzing the standard solutions, the Color Grab app (available for Android) must be downloaded. Thus, for a proper calibration curve acquisition, after all the preparation of the samples and thermal digestion treatment for COD analysis, according to the specific method described above, follow the next steps:

1. After cooling at ambient temperature, clean the vials for the total removal of finger marks or other dirt on the glass surface;
  *Even small marks on the glass surface can interfere with the saturation value given by the app.*
2. Clean the camera lens every time before analysis to guarantee camera accuracy.
  *In the same way as the glass vials, any dirt in the camera lens (inside or outside) can negatively affect the results of color saturation. At the slightest sign of loss of the smartphone camera capacity to perform clean and faithful results, the smartphone should be changed. To avoid problems like this, it is strongly recommended that more than one smartphone to be used to perform the calibration curve to compare the data. The best outlook is that every person that works in the group/laboratory performs their own curves with their smartphones.*
3. Position the sample near a white background;
  *The white background is prepared/used to guarantee the same conditions for the image capture.*
4. Use always artificial illumination for a proper standardization of the conditions;
  *Even a very lit ambient, it is passive for changes in the illumination throughout the day, for this reason, the only way to ensure the same conditions is with the use of the same artificial illumination.*
5. With the help of a ruler, take note of the position of the samples: the distance between the white background and the sample, and also between the sample and the smartphone;
  *Note that these distance conditions have to be strictly the same with all samples (2* *cm from the background to the sample, and more 5* *cm from the sample to the smartphone camera) in order to avoid deviations in the images capture.*
6. Open the Color Grab app and orientate the focus of the camera to the sample to be analyzed. The yellow circle that appears in the screen is the area to be analyzed (Fig. 1);
  *The Color Grab app has a didactic interface and it is very simple to use. Basically, in the moment in which the user opens the app and it immediately focuses on a determined surface, then, the app informs the HSV values of it, almost instantly, besides other information.*

**Fig. 1:**
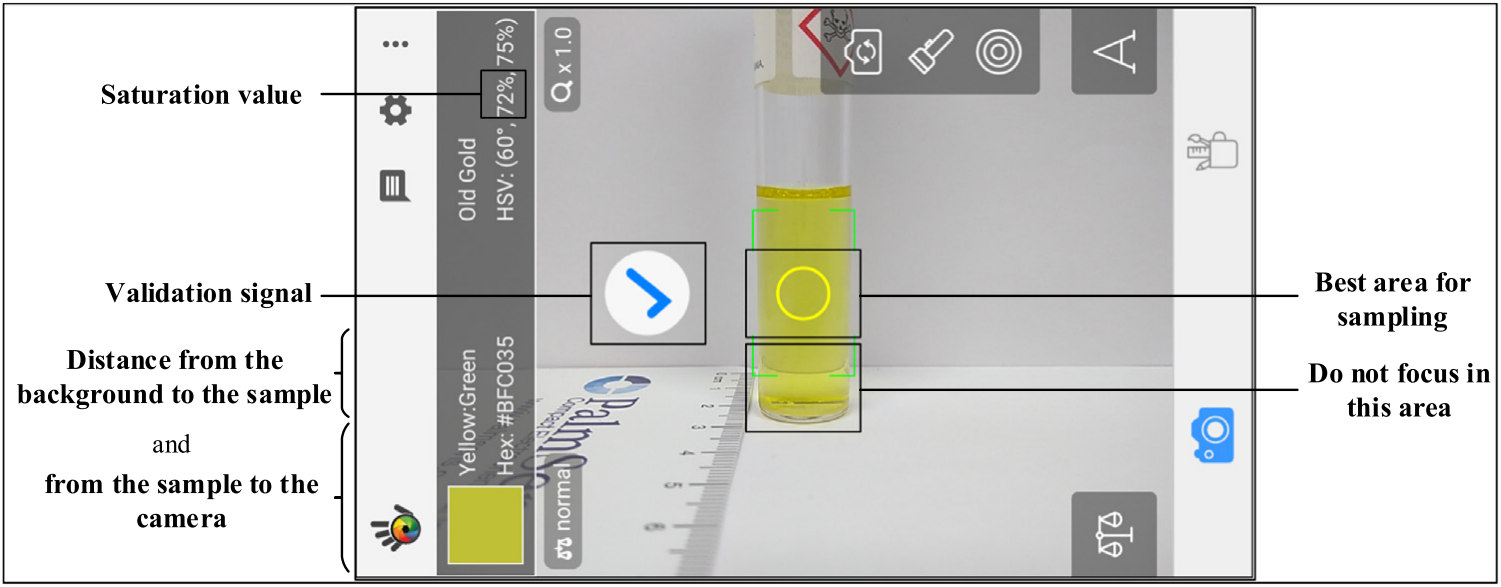
App appearance and main information of the image capture method.
7. After the validation signal appears (as shown in Fig. 1), a simple touch on the screen of the smartphone permits the capture of the necessary data, which is automatically saved;
  Fig. 1*points out the best area for sampling: in the center of the sample and avoiding the shadow area caused by the proximity of the background.*
8. Repeat the procedure with all the samples, taking note of all the saturation values necessary to construct the calibration curve.
9. Plot a graphic with the color saturation values against the theoretical COD (Fig. 2) values;
  *Use the data acquired with duplicates or triplicates to make the best calibration curve and further statistical analysis.*

**Fig. 2:**
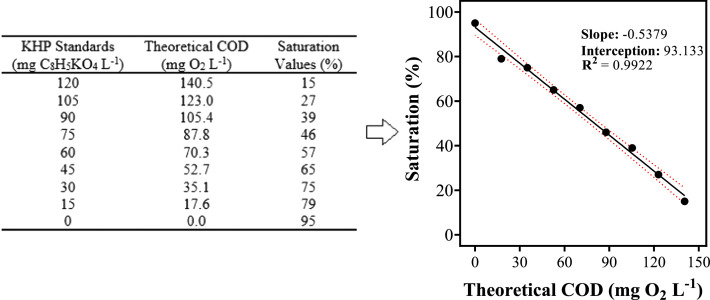
Graphical representation of the color saturation values of KHP standards against its theoretical COD. Confidence levels and standard deviations within 95% are indicated by red dashed lines on the plot. Thus, this information was used in order to identify false positives and false negatives (α = β = 0.05), as already reported by experts in the field [10,13].

10. With the equation of the straight line, it is possible to calculate and determine the COD value of the sample;
  *With an r^2^ higher than 0.99 is possible to guarantee a reliable result of COD concentration with the use of the equation of the straight line.*

It was proved in previous work [1] that the present method achieve 97% of average accuracy, demonstrating that no significant statistical differences were attained when compared to the results from the spectrophotometer. The research was performed for the first time for COD analysis with the raw effluent samples and also synthetic ones, and also by treating electrochemically them, making sure that real water matrices with substantial organic load and salts content can be analyzed by this innovative protocol [14].

## Conclusion

The linearity of the saturation values of the KHP standard solutions from COD measurements, based on the use of a simple smartphone with a camera, can be a promising way for environmental analysis when spectrophotometers are not available, such as decentralized approaches [15,16]. If correctly followed, this method proved to be statistically accurate in its measurements, according to the data obtained and estimated here as well as supported by the data in a previous work [1]. The method can also perform good opportunities for several laboratories and public or private organizations that deal with water quality and sanitation [15].

## CRediT authorship contribution statement

**Inalmar D. Barbosa Segundo:** Writing – original draft, Methodology, Investigation, Validation, Formal analysis. **Jussara C. Cardozo:** Methodology, Investigation. **Pollyana Souza Castro:** Investigation. **Amanda D. Gondim:** Resources, Funding acquisition. **Elisama V. dos Santos:** Resources, Funding acquisition. **Carlos A. Martínez-Huitle:** Conceptualization, Supervision, Writing – review & editing, Resources, Funding acquisition.

## Declaration of Competing Interest

The authors declare that they have no known competing financial interests or personal relationships that could have appeared to influence the work reported in this paper.

## Data Availability

The data that has been used is confidential.
